# Author Correction: Comprehensive characterization of 536 patient-derived xenograft models prioritizes candidates for targeted treatment

**DOI:** 10.1038/s41467-021-27678-7

**Published:** 2022-01-07

**Authors:** Hua Sun, Song Cao, R. Jay Mashl, Chia-Kuei Mo, Simone Zaccaria, Michael C. Wendl, Sherri R. Davies, Matthew H. Bailey, Tina M. Primeau, Jeremy Hoog, Jacqueline L. Mudd, Dennis A. Dean, Rajesh Patidar, Li Chen, Matthew A. Wyczalkowski, Reyka G. Jayasinghe, Fernanda Martins Rodrigues, Nadezhda V. Terekhanova, Yize Li, Kian-Huat Lim, Andrea Wang-Gillam, Brian A. Van Tine, Cynthia X. Ma, Rebecca Aft, Katherine C. Fuh, Julie K. Schwarz, Jose P. Zevallos, Sidharth V. Puram, John F. Dipersio, The NCI PDXNet Consortium, Julie Belmar, Jason Held, Jingqin Luo, Brian A. Van Tine, Rose Tipton, Yige Wu, Lijun Yao, Daniel Cui Zhou, Andrew Butterfield, Zhengtao Chu, Maihi Fujita, Chieh-Hsiang Yang, Emilio Cortes-Sanchez, Sandra Scherer, Ling Zhao, Tijana Borovski, Vicki Chin, John DiGiovanna, Christian Frech, Jeffrey Grover, Ryan Jeon, Soner Koc, Jelena Randjelovic, Sara Seepo, Tamara Stankovic, Lacey E. Dobrolecki, Michael Ittmann, Susan G. Hilsenbeck, Bert W. O’Malley, Nicholas Mitsiades, Salma Kaochar, Argun Akcakanat, Jithesh Augustine, Huiqin Chen, Bingbing Dai, Kurt W. Evans, Kelly Gale, Don Gibbons, Min Jin Ha, Vanessa Jensen, Michael Kim, Bryce P. Kirby, Scott Kopetz, Christopher D. Lanier, Dali Li, Mourad Majidi, David Menter, Ismail Meraz, Turcin Saridogan, Stephen Scott, Alexey Sorokin, Coya Tapia, Jing Wang, Shannon Westin, Yuanxin Xi, Yi Xu, Fei Yang, Timothy A. Yap, Vashisht G. Yennu-Nanda, Erkan Yuca, Jianhua Zhang, Ran Zhang, Xiaoshan Zhang, Xiaofeng Zheng, Dylan Fingerman, Haiyin Lin, Qin Liu, Andrew V. Kossenkov, Vito W. Rebecca, Rajasekharan Somasundaram, Michae T. Tetzlaff, Jayamanna Wickramasinghe, Min Xiao, Xiaowei Xu, Carol J. Bult, Peter N. Robinson, Anuj Srivastava, Michael W. Lloyd, Steven B. Neuhauser, Jill Rubinstein, Brian J. Sanderson, Brian White, Xing Yi Woo, Tiffany Wallace, John D. Minna, Gao Boning, Luc Girard, Hyunsil Park, Brenda C. Timmons, Katherine L. Nathanson, George Xu, Chong-xian Pan, Moon S. Chen Jr, Luis G. Carvajal-Carmona, May Cho, Nicole B. Coggins, Ralph W. deVere White, Guadalupe Polanco-Echeverry, Ana Estrada, David R. Gandara, Amanda R. Kirane, Tiffany Le, Paul Lott, Alexa Morales Arana, Jonathan W. Reiss, Sienna Rocha, Clifford G. Tepper, Ted Toal, Hongyong Zhang, Ai-Hong Ma, Brandi Davis-Dusenbery, Matthew J. Ellis, Michael T. Lewis, Michael A. Davies, Meenhard Herlyn, Bingliang Fang, Jack A. Roth, Alana L. Welm, Bryan E. Welm, Funda Meric-Bernstam, Feng Chen, Ryan C. Fields, Shunqiang Li, Ramaswamy Govindan, James H. Doroshow, Jeffrey A. Moscow, Yvonne A. Evrard, Jeffrey H. Chuang, Benjamin J. Raphael, Li Ding

**Affiliations:** 1 Department of Medicine, Washington University in St. Louis, St. Louis, MO USA; 2 McDonnell Genome Institute, Washington University in St. Louis, St. Louis, MO USA; 3 Department of Computer Science, Princeton University, Princeton, NJ USA; 4 Computational Cancer Genomics Research Group and Cancer Research UK Lung Cancer Centre of Excellence, University College London Cancer Institute, London, UK; 5 Department of Mathematics, Washington University in St. Louis, St. Louis, MO USA; 6 Department of Genetics, Washington University in St. Louis, St. Louis, MO USA; 7 Huntsman Cancer Institute, University of Utah, Salt Lake City, UT USA; 8 Seven Bridges Genomics, Inc., Cambridge, Charlestown, MA USA; 9 Frederick National Laboratory for Cancer Research, Frederick, MD USA; 10 Siteman Cancer Center, Washington University in St. Louis, St. Louis, MO USA; 11 Department of Radiation Oncology, Washington University in St. Louis, St. Louis, MO USA; 12 Department of Otolaryngology, Washington University St. Louis, St. Louis, MO USA; 13 Lester and Sue Smith Breast Center, Baylor College of Medicine, Houston, TX USA; 14 The University of Texas MD Anderson Cancer Center, Houston, TX USA; 15 The Wistar Institute, Philadelphia, PA USA; 16 Division of Cancer Treatment and Diagnosis, National Cancer Institute, Bethesda, MD USA; 17 Investigational Drug Branch, National Cancer Institute, Bethesda, MD USA; 18 The Jackson Laboratory for Genomic Medicine, Farmington, CT USA; 19 Department of Pathology, Baylor College of Medicine, Houston, TX USA; 20 Department of Molecular and Cellular Biology, Baylor College of Medicine, Houston, TX USA; 21 Department of Medicine, Baylor College of Medicine, Houston, TX USA; 22 Center to Reduce Cancer Health Disparities, National Cancer Institute, Bethesda, MD USA; 23 Hamon Center for Therapeutic Oncology, UT Southwestern Medical Center, Dallas, TX USA; 24 Abramson Cancer Center, University of Pennsylvania, Philadelphia, PA USA; 25 Department of Pathology and Laboratory Medicine, Hospital of the University of Pennsylvania, Philadelphia, PA USA; 26 University of California Davis, Sacramento, CA USA

**Keywords:** Cancer genomics, Cancer models, Data integration, Pharmacogenomics

Correction to: *Nature Communications* 10.1038/s41467-021-25177-3, published online 24 August 2021.

In this Article the Competing interests statement inadvertently omitted a statement for M.T.L. and L.E.D. The competing financial interests for M.T.L. should have read: ‘M.T.L. is a founder and limited partner in StemMed Ltd. and a manager in StemMed Holdings, its general partner. He is a founder and equity stakeholder in Tvardi Theraeutics Inc. Some PDXs are exclusively licensed to StemMed Ltd. resulting in royalty income to M.T.L.’. The competing financial interests for L.E.D. should have read, ‘L.E.D. is a compensated employee of StemMed Ltd’. The PDF and HTML versions of the Article have been corrected.

