# Supplementary material for: Comprehensive characterization of 536 patient-derived xenograft models prioritizes candidates for targeted treatment
Source: Nat Commun. 2021 Aug 24;12:5086. doi: 10.1038/s41467-021-25177-3 (PMC8384880; doi:10.1038/s41467-021-25177-3)
Supplement: Supplementary file 1 — Supplementary Information [file 41467_2021_25177_MOESM1_ESM.pdf]

SUPPLEMENTARY INFORMATION

Comprehensive characterization of 536 patient-derived xenograft models prioritizes candidates for targeted treatment

CONTENTS

Supplementary Methods

Supplementary Figures

Supplementary References

SUPPLEMENTARY METHODS

Washington University in St. Louis (WUSTL)*.* All human tissues acquired for experiments were processed in compliance with NIH regulations and institutional guidelines, as approved by the Institutional Review Board at WUSTL. All tumor materials from patients were obtained either via core needle biopsy, skin punch biopsy, or surgical resection after informed consent. All animal procedures were reviewed by and received ethical approval from the Institutional Animal Care and Use Committee at WUSTL. Pancreatic cancer models were derived from tissue fragments implanted subcutaneously into dorsal flank regions of 5-week-old non-humanized, female NOD/SCID/γ mice (Jackson Laboratory, Bar Harbor, ME) using Matrigel. Animal environments were maintained at 68-72°F and 45-55% relative humidity. The sample tissues for these PDX models were obtained from archived, cryopreserved PDX harvests. Final tumor passages in mice were kept cold and harvested into RPMI-1640 with antibiotic and antimycotic additives. Patient tumors were obtained directly from operating rooms and placed into sterile collection media (RPMI-1640 with antibiotic and antimycotic additives). Pieces of each tumor were processed into the following forms: flash frozen tissue fragments, OCT blocks and matched haemotoxylin and eosin (H&E) slides, formalin fixed paraffin blocks (FFPE) and matched H&E slides, RNA later tissue storage, and cryopreserved fragments (FBS + 10% DMSO). A minimum of 250 mg of flash frozen material was submitted to the Siteman Cancer Center's Proteomics Core. The tissues were cryo-pulverized and subsequently divided for DNA and RNA preparation and long-term storage. Parental genomic DNA was prepared from OCT blocks if available, and if not available, paraffin blocks were utilized. In addition, genomic DNA for sequencing control was prepared from peripheral blood mononuclear cells. Whole-exome sequencing was conducted as follows: Libraries were constructed using unamplified genomic DNA (minimum 100 ng) from blood (normal), tumor, and xenograft samples. Exons were captured via IDT Exome library kit followed by high-throughput sequencing on an Illumina NovaSeq S4 platform (Illumina Inc., San Diego, CA) using 150bp paired-end reads. RNA-seq was performed using NovaSeq 6000 sequencing system.

National Cancer Institute Patient-Derived Models Repository (PDMR). Solid tumor specimens across histologies are collected from patients being evaluated for and/or treated for cancer at clinical centers across the United States under National Cancer Institute (NCI)-sponsored tissue procurement protocols with institutional review board approval. Exclusion criteria were limited to certain infections that would compromise either the ability to make or to distribute preclinical models by the NCI. Investigators obtained informed consent/assent from each participant for the use of their delinked specimens specifically to generate and genetically characterize animal models and cell cultures and to have these models made available to researchers along with limited information about their medical history, also delinked, through a public database (https://pdmr.cancer.gov/). Information about which clinical center and which investigator provided the specimen are not recorded. Models for distribution by NCI's

Patient-Derived Models Repository were generated at the Biological Testing Branch, DTP, NCI-Frederick. NCI-Frederick is accredited by the Association for Assessment and Accreditation of Laboratory Animal Care International and follows the Public Health Service (PHS) Policy on Humane Care and Use of Laboratory Animals. All studies were conducted on an approved NCI at Frederick Institutional Animal Care and Use Committee protocol in accordance with the procedures outlined in the "Guide for Care and Use of Laboratory Animals" (National Research Council; 2011; National Academy Press; Washington, DC). Mice were housed in sterile, filter-capped polycarbonate cages, maintained in a barrier facility on a 12-hour light/dark cycle, and were provided sterilized food and water, ad libitum. For engraftments, tumor material plus a drop of Matrigel (BD BioSciences, Bedford, MA) were implanted subcutaneously in NOD.Cg-*Prkdc*^{scid} *Il2rg*^{tm1Wjl}/SzJ (NSG) mice that are sex-matched to the human patient. Animals were monitored weekly for tumor growth. The initial passage of material was grown to approximately 1000-2000 mm^3 calculated using the following formula: weight (mg) = (tumor length × [tumor width]^2) / 2. Tumor material was then harvested, a portion cryopreserved, and the remainder implanted into NSG host mice. Every PDX tumor harvested and cryopreserved also had 2-3 fragments snap frozen for next generation sequence analysis and short tandem repeat validation, as well as a piece fixed in neutral buffered formalin and then embedded in paraffin for histological assessment. Full PDMR standard operating procedures for tumor engraftment and PDX passaging are available at https://pdmr.cancer.gov/sops. The whole-exome sequencing was conducted based on PDMR guidelines available online at https://pdmr.cancer.gov/content/docs/MCCRD_SOP0009_PDX_Illumina_Sequencing_library_pools_external.pdf. The RNA-Seq library preparation and sequencing were performed based on PDMR guidelines available at https://pdmr.cancer.gov/content/docs/MCCRD_SOP0006_PDX_Automated_RNAAccess_LibraryPrep_Illumina.pdf.

The University of Texas MD Anderson Cancer Center (MDACC)*.* Fresh non-small-cell lung carcinoma tumor samples were collected from surgically resected specimens with the informed consent of the patients. The sex and race distributions of patients were similar to those of patient throughout the USA. Generation and passaging of PDXs, and histological analysis and DNA fingerprint assay for PDXs and their primary tumor tissues were performed as previously described [1,2] . Early PDXs were generated and passaged in 6-10 week-old NOD/SCID mice, while later PDXs were generated using NSG mice and then passaged solely in nude mice (80% success rate) or otherwise solely in NSG mice. Both male and female mice were used in the study. The protocols for the use of clinical specimens and data in this study were approved by the Institutional Review Board at The University of Texas MD Anderson Cancer Center. All animal studies were carried out in accordance with the Guidelines for the Care and Use of Laboratory Animals (National Institutes of Health Publication 85-23) and the institutional guidelines of MDACC. Mice were housed in 70-74°F rooms at 40-55% relative humidity. Whole-exome sequencing followed MDACC protocol [3]. Equimolar amounts of DNA were pooled (2-6 samples per pool) and whole exome regions were captured by using biotin labeled probes from Roche Nimblegen (Exome V3) followed

manufacturer's protocol. The captured libraries were sequenced on a HiSeq 2000 with 100bp paired-end (Illumina Inc., San Diego, CA, USA) on a paired-end flowcell.

The WISTAR Institute (WISTAR*).* Tumor biopsy samples were collected with the informed, written consent of the patients according to a Wistar Institutional Review Board (IRB) approved protocol. Animal studies were carried out in accordance with the Wistar Animal Care and Use Program as overseen by The Wistar Institutional Animal Care and Use Committee (IACUC). The protocol to generate PDX models requires the subcutaneous implantation of fresh tissue from primary or metastatic melanomas (collected by biopsy or surgery) into NOD/SCID/IL2-receptor null (NSG) mice. Detailed preparation guidelines are given by Xiao et al. [4] and Krepler et al. [5] Whole exome sequencing was conducted as follows: Genome DNA extraction was done using Qiagen DNeasy Blood & Tissue Kit, and libraries for whole exome sequencing were performed using Nextera DNA exome kit. Capture libraries were amplified, pooled, and then sequenced on an Illumina HiSeq 2500 76bp paired-end run. Average coverage for normal samples was 97.50x (71.46 min – 124.64 max), and was 208.27x for tumor samples (146.88 min – 281.20 max). More details of the standard animal and genomics protocols of WISTAR is available at https://wistar.org/research-discoveries/shared-resources/.

Baylor College of Medicine (BCM). Human breast cancers were procured as stably transplantable xenografts previously established in SCID/Beige or NOD/SCID IL2Rgamma-null (NSG) immunocompromised mice [6, 7]. PDX lines were maintained by serial transplantation into the inguinal mammary fat pad into 3-5 week-old female mice of the aforementioned strains using jeweler's forceps via a 0.5 cm incision in the skin of the animal to expose the fat pad. Incisions were closed with a single staple and animals allowed to recover on a warming plate. Animal environments were maintained at 68-72°F at 30-70% relative humidity under a 10:14 130-300 lux light cycle with standard breeding and regular diets provided. These xenografts represent the major clinically defined subtypes of breast cancer, e.g. estrogen receptor positive (ER+), HER2 positive (HER2+), and "triple negative" (TN) breast cancers. The detailed methods of implantation and strains were presented by Zhang and Lewis [8]. For that work, breast cancer patients were recruited from clinics in the Baylor College of Medicine Breast Center and Ben Taub General Hospital under IRB-approved protocols, which included informed, written patient consent. For ER+ PDX lines, estradiol (8ug/ml) was provided in the drinking water. Animal care was in accordance with the NIH Guide for the Care and Use of Experimental Animals with approval from the Baylor College of Medicine Institutional Animal Care and Use Committee and in compliance with all relevant ethical regulations for animal testing and research. Preparation and generation of whole exome and RNA-Seq data were undertaken as previously reported [9, 10].

Huntsman Cancer Institute (HCI). All tissue samples were collected with informed consent from individuals being treated at the Huntsman Cancer Hospital and the University of Utah under a protocol approved by the University of Utah Institutional Review Board. Samples were collected and de-identified by the Huntsman Cancer Institute Tissue Resource and Application Core facility before being obtained for

implantation. Tumor processing, histology, and immunostaining were described by DeRose et al. [11] Animal husbandry was in accordance with the University of Utah and Animal Welfare Act, and all mouse studies were reviewed and approved by the University of Utah's Office of Institutional Animal Care and Use Committee (IACUC). Detailed methods of implantation into 3- to 4-week-old female NOD/SCID mice are presented by DeRose et al. [11] and DeRose et al. [12]. Details of sequencing methods were described by Ma et al., 2020 (doi: https://doi.org/10.1101/2020.06.25.172056) and Woo et al., 2021 [3].

SUPPLEMENTARY FIGURES

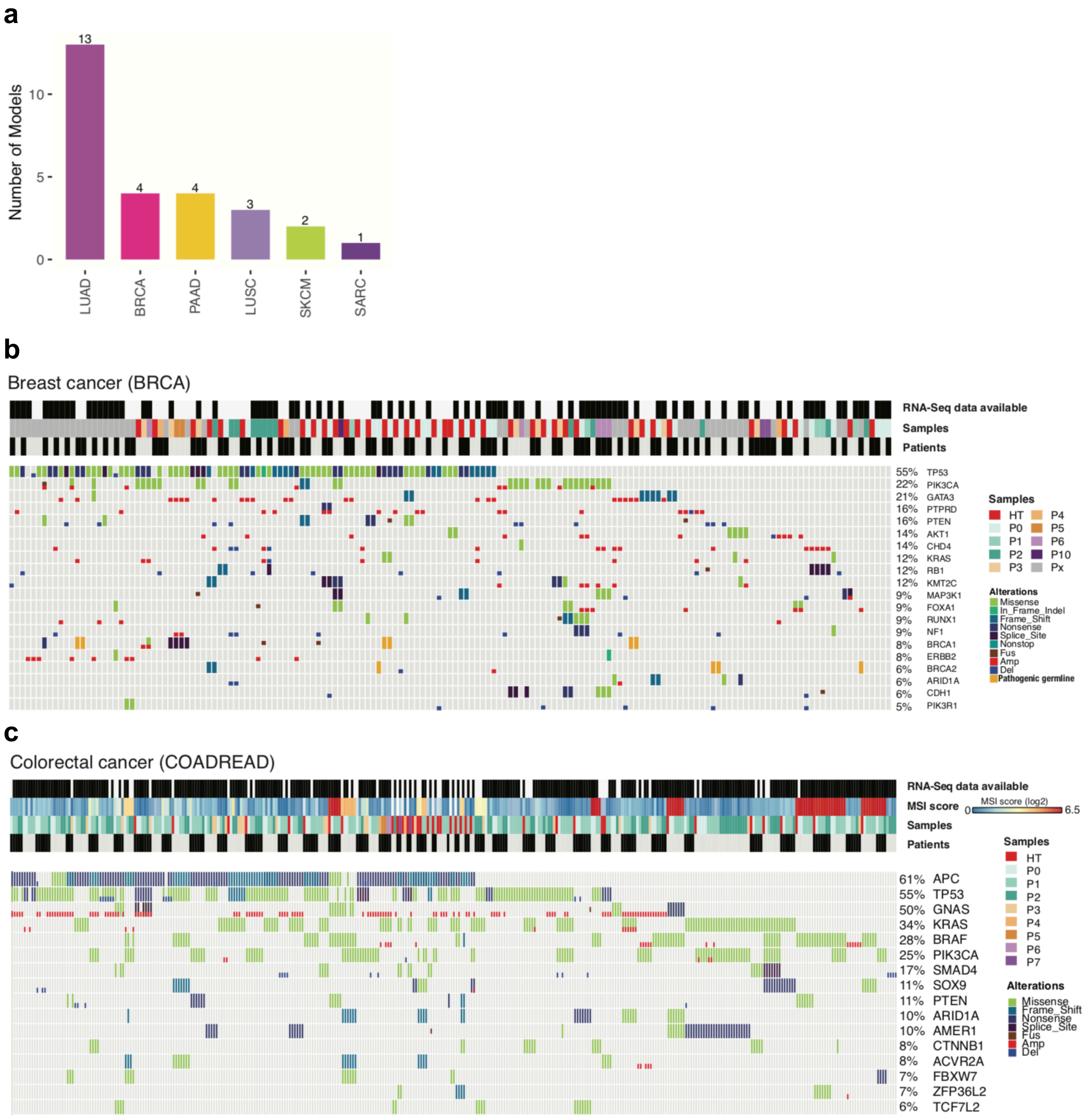

Supplementary Fig. 1: a The number of models per cancer type for low similarity events. Genetic alterations found in (**b**) 97 BRCA patients, and (**c**) 72 COADREAD patients. Source data are provided as a Source Data file.

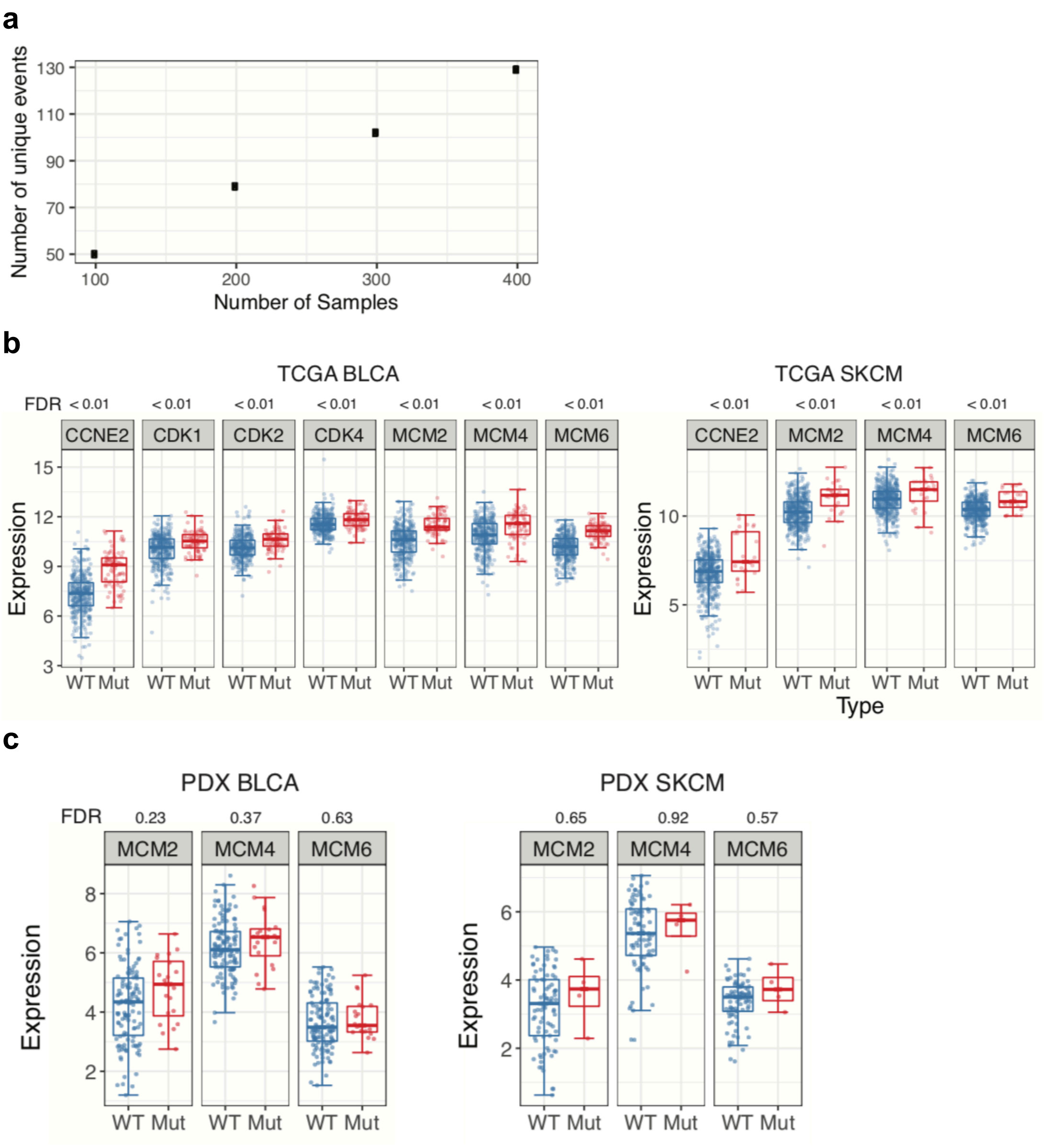

Supplementary Fig. 2: a The dependence of unique cis and trans events on sample size for BLCA. **b** The effects of *RB1* mutations on the expression of *CDK*, *CCNE*, and *MCM* genes based on TCGA data. Sample numbers (n) for (WT, Mut) are (330, 76) for BLCA and (439, 26) for SKCM **c** The comparison of *CDK*, *CCNE*, and *MCM* gene expression between mutated and WT-type *RB1* PDX samples. Sample numbers (n) for (WT, Mut) are (120, 20) for BLCA and (81,4) for SKCM. Source data are provided as a Source Data file. The box boundary of each box plot indicates third quartile and first quartile respectively from the top to bottom. The whisker on top were drawn out from the third quartile to the largest data point or up to 1.5 × IQR. Similarly, the bottom whisker extends from the first quartile down to 1.5 × IQR or the lowest data point. The red dot at the center indicates medium. Source data are provided as a Source Data file.

a

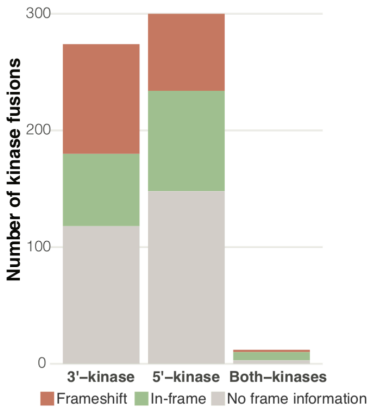

b

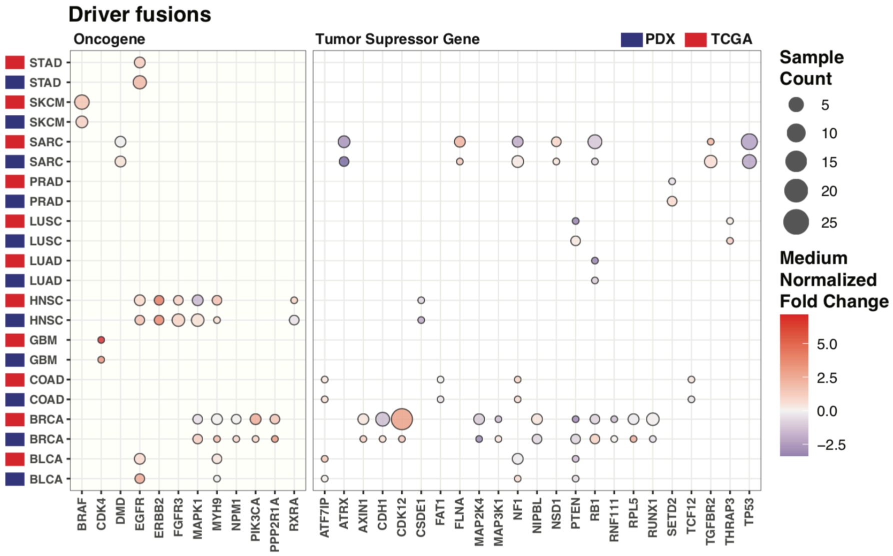

Supplementary Fig. 3: a Distribution of kinase fusions based on kinase locations (5'-end, 3'-end, or as both fusion partners) and the in-frame status. **b** Median normalized expression of fusion involving oncogenes and tumor suppressor genes per cancer type. Fusion in greater than 2 PDX samples while overlap with TCGA events are shown. Source data are provided as a Source Data file.

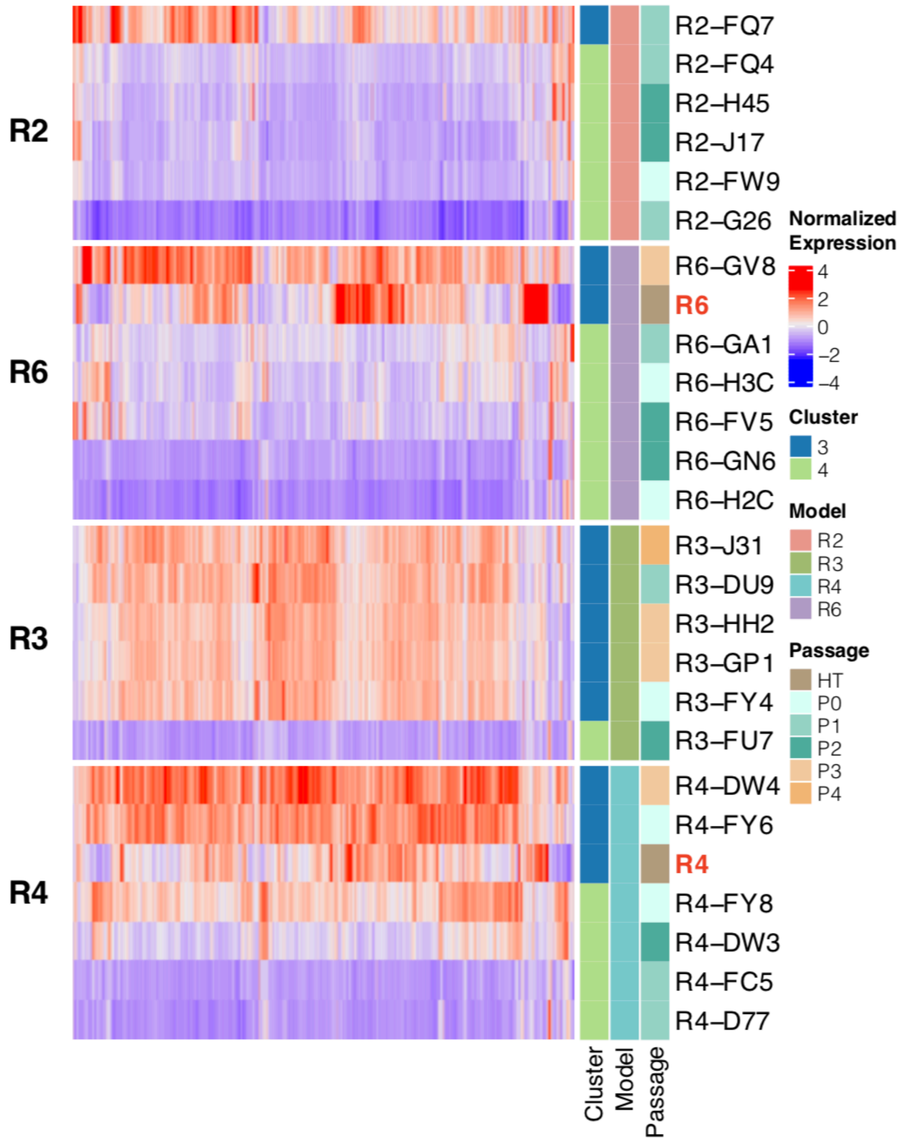

Supplementary Fig. 4: RNA expression for the top 1000 most variable genes in 4 PDX models (R2, R3, R4 and R6) from case PDMR-521955. Human tumor samples highlighted in red. Source data are provided as a Source Data file.

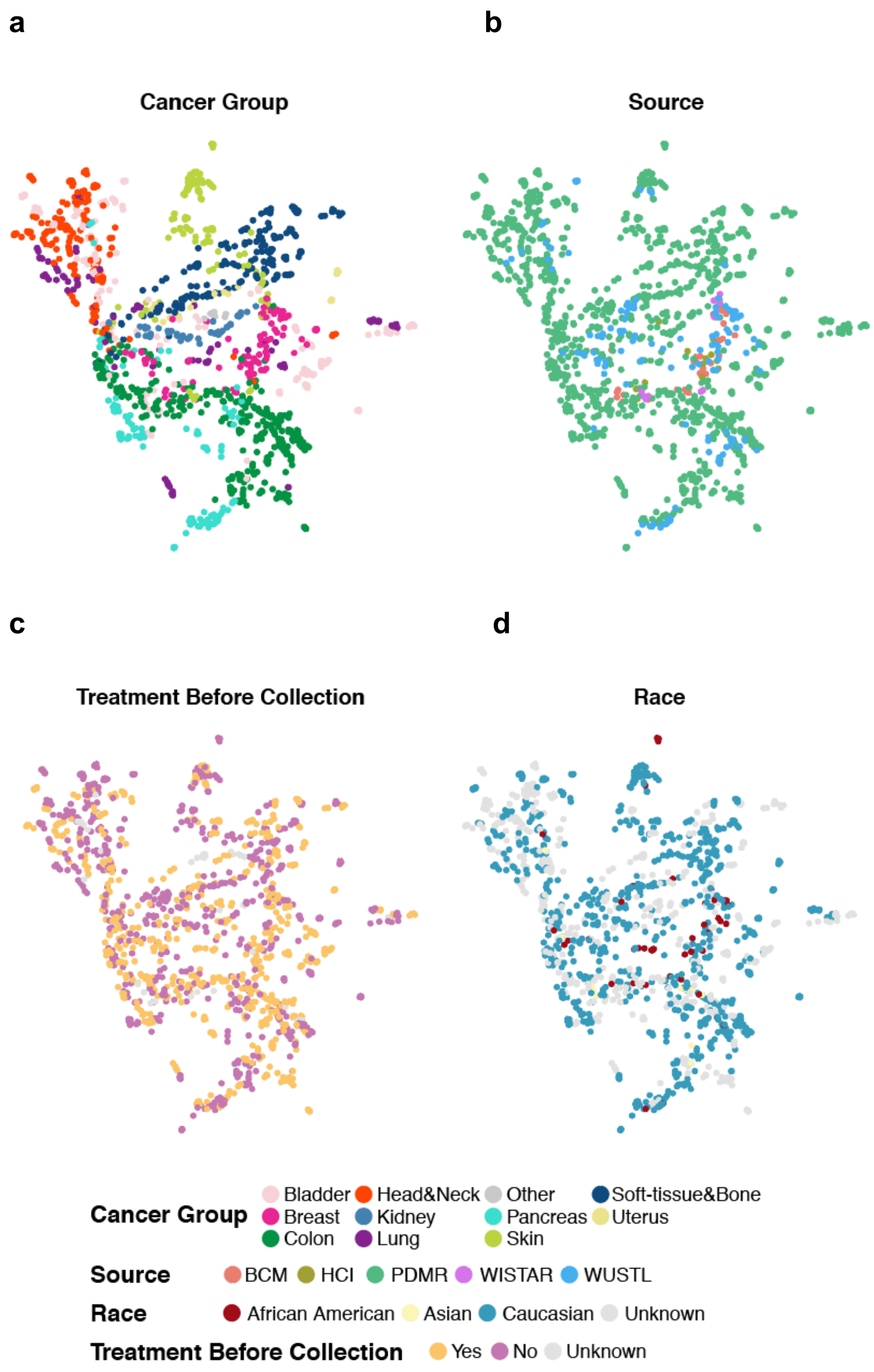

Supplementary Fig. 5: Additional pan-cancer transcriptional group distribution on 2D UMAP. 2D UMAP distributions of (**a**) cancer groups, (**b**) collection center, (**c**) treatment status before collection, and (**d**) racial group. Source data are provided as a Source Data file.

a

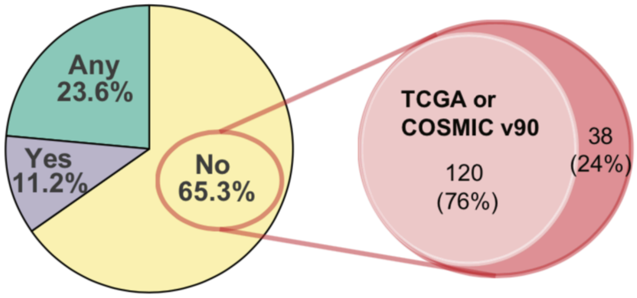

b

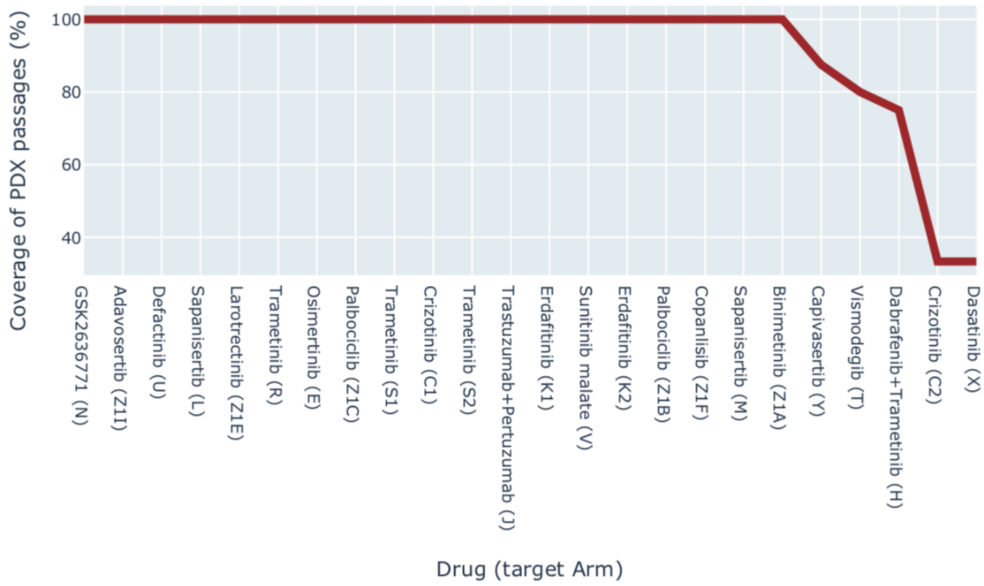

Supplementary Fig. 6: Comparison of genetic alterations with DEPO and CIViC databases and estimation of arm coverages across PDX passages. **a** Percentage of known druggable gene alteration in PDX genetic alterations. ‘Yes’ and ‘Any’ mean that the alterations have known drugs, and ‘No’ means there are no known drugs that match with the genetic alterations. The Venn diagram presents that 76% genetic alterations matched with TCGA or COMIC (v90) database, and 24% genetic alterations belong to unknown alterations. **b** Display positive signals for the target arms via the percentage of PDX passages. Source data are provided as a Source Data file.

SUPPLEMENTARY REFERENCES

1. Chen Y*, et al.* Tumor characteristics associated with engraftment of patient-derived non-small cell lung cancer xenografts in immunocompromised mice. *Cancer* **125**, 3738-3748 (2019).

2. Hao C*, et al.* Gene mutations in primary tumors and corresponding patient-derived xenografts derived from non-small cell lung cancer. *Cancer Lett* **357**, 179-185 (2015).

3. Woo XY*, et al.* Conservation of copy number profiles during engraftment and passaging of patient-derived cancer xenografts. *Nat Genet* **53**, 86-99 (2021).

4. Xiao M, Rebecca VW, Herlyn M. A Melanoma Patient-Derived Xenograft Model. *J Vis Exp*, (2019).

5. Krepler C*, et al.* Personalized Preclinical Trials in BRAF Inhibitor-Resistant Patient-Derived Xenograft Models Identify Second-Line Combination Therapies. *Clin Cancer Res* **22**, 1592-1602 (2016).

6. Clarke R. Animal models of breast cancer: their diversity and role in biomedical research. *Breast Cancer Res Treat* **39**, 1-6 (1996).

7. Ramirez AB*, et al.* Circulating tumor cell investigation in breast cancer patient-derived xenograft models by automated immunofluorescence staining, image acquisition, and single cell retrieval and analysis. *BMC Cancer* **19**, 220 (2019).

8. Zhang X, Lewis MT. Establishment of Patient-Derived Xenograft (PDX) Models of Human Breast Cancer. *Curr Protoc Mouse Biol* **3**, 21-29 (2013).

9. Rokita JL*, et al.* Genomic Profiling of Childhood Tumor Patient-Derived Xenograft Models to Enable Rational Clinical Trial Design. *Cell Rep* **29**, 1675-1689 (2019).

10. Neelakantan D*, et al.* EMT cells increase breast cancer metastasis via paracrine GLI activation in neighbouring tumour cells. *Nat Commun* **8**, 15773 (2017).

11. DeRose YS*, et al.* Tumor grafts derived from women with breast cancer authentically reflect tumor pathology, growth, metastasis and disease outcomes. *Nat Med* **17**, 1514-1520 (2011).

12. DeRose YS*, et al.* Patient-derived models of human breast cancer: protocols for in vitro and in vivo applications in tumor biology and translational medicine. *Curr Protoc Pharmacol* **Chapter 14**, Unit14 23 (2013).
