# Supplementary material for: Comprehensive characterization of 536 patient-derived xenograft models prioritizes candidates for targeted treatment
Source: Nat Commun. 2021 Aug 24;12:5086. doi: 10.1038/s41467-021-25177-3 (PMC8384880; doi:10.1038/s41467-021-25177-3)
Supplement: Supplementary file 2 — Reporting Summary [file 41467_2021_25177_MOESM2_ESM.pdf]

nature research

Corresponding author(s): Li Ding
Last updated by author(s): Jun 25, 2021

Reporting Summary

Nature Research wishes to improve the reproducibility of the work that we publish. This form provides structure for consistency and transparency in reporting. For further information on Nature Research policies, see our Editorial Policies and the Editorial Policy Checklist.

Statistics

For all statistical analyses, confirm that the following items are present in the figure legend, table legend, main text, or Methods section.

n/a	Confirmed	
[ ]	[x]	The exact sample size (n) for each experimental group/condition, given as a discrete number and unit of measurement
[ ]	[x]	A statement on whether measurements were taken from distinct samples or whether the same sample was measured repeatedly
[ ]	[x]	The statistical test(s) used AND whether they are one- or two-sided<br>*Only common tests should be described solely by name; describe more complex techniques in the Methods section.*
[x]	[ ]	A description of all covariates tested
[ ]	[x]	A description of any assumptions or corrections, such as tests of normality and adjustment for multiple comparisons
[x]	[ ]	A full description of the statistical parameters including central tendency (e.g. means) or other basic estimates (e.g. regression coefficient) AND variation (e.g. standard deviation) or associated estimates of uncertainty (e.g. confidence intervals)
[ ]	[x]	For null hypothesis testing, the test statistic (e.g. F, t, r) with confidence intervals, effect sizes, degrees of freedom and P value noted<br>*Give P values as exact values whenever suitable.*
[x]	[ ]	For Bayesian analysis, information on the choice of priors and Markov chain Monte Carlo settings
[x]	[ ]	For hierarchical and complex designs, identification of the appropriate level for tests and full reporting of outcomes
[x]	[ ]	Estimates of effect sizes (e.g. Cohen's d, Pearson's r), indicating how they were calculated

Our web collection on statistics for biologists contains articles on many of the points above.

Software and code

Policy information about availability of computer code

Data collection	Sequence files were collected from all sources using the freely available GNU Wget [www.gnu.org/software/wget/] software (version 1.14). Additional data was obtained from on-line resources as CSV exports or as Excel spreadsheets, which were subsequently converted to CSV for downstream processing.
Data analysis	Full descriptions of all analysis techniques are provided in the Methods. Software/tools/packages include R v3.6, SIFT v5.2.2, PolyPhen v2.2.2, Trim Galore v0.5.0, Samtools v1.5, Picard v2.20.1, BWA-MEM v0.7.17, Disambiguate v1.0, SeqQEst beta version, SomaticWrapper v1.5, Strelka v2.9.2, VarScan v2.3.8, Pindel v0.2.5, MuTect v1.1.7, bam-readcount v0.7, Mutect2 v4.1.2.0, GermlineWrapper v1.1, GATK v4.0, CharGer v0.5.4, ClinVar v08/15/2019, CNVKit v0.96, Gistic2.0, HATCHet v0.1, Kallisto v0.44, tximport v1.12.0, ABSOLUTE v1.0.6, ESTIMATE v2.0, STAR-Fusion v1.6.0, MSIsensor v0.6, MSIsensor2 v0.1<br>Source code for data processing and analysis are available from the GitHub repository: https://github.com/ding-lab/PDX-PanCanAtlas<br>SeqQEst (https://github.com/ding-lab/SeqQEst)<br>SomaticWrapper (https://github.com/ding-lab/somaticwrapper)<br>Mutect2_tumorOnly (https://github.com/ding-lab/PDX-PanCanAtlas/tree/master/data_process/somatic.Mutect2_tumorOnly)

For manuscripts utilizing custom algorithms or software that are central to the research but not yet described in published literature, software must be made available to editors and reviewers. We strongly encourage code deposition in a community repository (e.g. GitHub). See the Nature Research guidelines for submitting code & software for further information.

Data

Policy information about availability of data

All manuscripts must include a data availability statement. This statement should provide the following information, where applicable:

- Accession codes, unique identifiers, or web links for publicly available datasets
- A list of figures that have associated raw data
- A description of any restrictions on data availability

Clinical and sequence data, available through the National Cancer Institute (NCI) Cancer Data Service (https://datacommons.cancer.gov/repository/cancer-data-service), represent contributions from multiple sources, including the Washington University PDX Development and Trials Center, the University of Texas MD Anderson Cancer Center, Huntsman Cancer Institute, The WISTAR Institute, the Baylor College of Medicine, and the NCI Patient-Derived Models Repository (PDMR). Hyperlinks to these PDX centers and model descriptions are listed in Supplementary Data 6. Source data are provided with this paper. All available omics data are deposited at Figshare (https://doi.org/10.6084/m9.figshare.14390408) and have been reformatted for viewing through the "PDX Variant Viewer" web portal (https://pdx.wustl.edu/pdx). Published datasets used in this manuscript are available through the following website: GENCODE (https://www.gencodegenes.org); COSMIC (https://cancer.sanger.ac.uk/cosmic); TCGA-MC3 (https://gdc.cancer.gov/about-data/publications/mc3-2017); GDC panel-of-normals (PON) (https://gdc.cancer.gov/about-data/gdc-data-processing/gdc-reference-files); gnomAD (https://gnomad.broadinstitute.org); dbSNP (https://ftp.ncbi.nih.gov/snp/organisms/human_9606_b151_GRCh38p7); dbNSFP (https://sites.google.com/site/jpopgen/dbNSFP); CIViC (https://civicdb.org/home); DEPO (https://github.com/ding-lab/publicDEPO); NCI-MATCH/EAY131 Precision Medicine Trial (https://ecog-acrin.org/trials/nci-match-eay131).

Field-specific reporting

Please select the one below that is the best fit for your research. If you are not sure, read the appropriate sections before making your selection.

[x] Life sciences   [ ] Behavioural & social sciences   [ ] Ecological, evolutionary & environmental sciences

For a reference copy of the document with all sections, see nature.com/documents/nr-reporting-summary-flat.pdf

Life sciences study design

All studies must disclose on these points even when the disclosure is negative.

Sample size	We included 536 PDX models (comprised of 1,760 PDX samples) and 268 human tumors.
Data exclusions	Sequence files that passed our described QC method were used without further selection criteria.
Replication	All cis and trans effects observed in PDX in Figure 3 are validated by TCGA data.
Randomization	Analysis of PDX models is purely based on the data availability.
Blinding	The sample collection and generation are mostly from the PDXNet and PDMR consortia, and we are blind to sample collection.

Reporting for specific materials, systems and methods

We require information from authors about some types of materials, experimental systems and methods used in many studies. Here, indicate whether each material, system or method listed is relevant to your study. If you are not sure if a list item applies to your research, read the appropriate section before selecting a response.

Materials & experimental systems

n/a	Involved in the study
[x]	[ ] Antibodies
[x]	[ ] Eukaryotic cell lines
[x]	[ ] Palaeontology and archaeology
[ ]	[x] Animals and other organisms
[ ]	[x] Human research participants
[x]	[ ] Clinical data
[x]	[ ] Dual use research of concern

Methods

n/a	Involved in the study
[x]	[ ] ChIP-seq
[x]	[ ] Flow cytometry
[x]	[ ] MRI-based neuroimaging

Animals and other organisms

Policy information about studies involving animals; ARRIVE guidelines recommended for reporting animal research

Laboratory animals	Details on mouse strains used for patient-derived xenograft studies are provided in the Methods and Supplementary information.
Wild animals	No wild animals

Field-collected samples	No field-collected samples
Ethics oversight	This study is a meta-analysis of data collected across a large number of consortium sites and does not follow a single study protocol. All studies were conducted in compliance with ethics regulations, as detailed in the Methods and Supplementary information.

Note that full information on the approval of the study protocol must also be provided in the manuscript.

Human research participants

Policy information about studies involving human research participants

Population characteristics	The population characteristics of the participants that the PDX models in this study were derived from are included in the table in Supplementary Data 1. Since this is a meta-analysis of PDX tumor sequence samples contributed by multiple institutions under different protocols, population characteristics are relatively undefined. Tumors were collected under informed consent from participants with solid tumor malignancies at various stages of disease. The ages of diagnosis among participants ranged 17-91 years, and the sex distribution was 46% female and 54% male.
Recruitment	The participant populations that were used to develop the PDX models analyzed in this study varied across multiple consortium sites with different protocols. The primary common inclusion criteria were that the participants had solid tumor malignancies where samples could be collected under informed consent for research and PDX development.
Ethics oversight	This study is a meta-analysis of data collected across a large number of consortium sites and does not follow a single study protocol. All studies were conducted in compliance with ethics regulations, as detailed in the Methods and Supplementary information.

Note that full information on the approval of the study protocol must also be provided in the manuscript.
