# Supplementary material for: Comprehensive characterization of 536 patient-derived xenograft models prioritizes candidates for targeted treatment
Source: Nat Commun. 2021 Aug 24;12:5086. doi: 10.1038/s41467-021-25177-3 (PMC8384880; doi:10.1038/s41467-021-25177-3)
Supplement: Supplementary file 4 — Description of Additional Supplementary Files [file 41467_2021_25177_MOESM4_ESM.pdf]

Description of Additional Supplementary Files

File Name: Supplementary Data 1
Description: Data and clinical information including cancer type, PDX model, passage, clinical info, sequence QC, tumor purity, MSI score and so on.

File Name: Supplementary Data 2.
Description: Model ids, cancer type, similarity score, germlineQC score for 27 low similarity models.

File Name: Supplementary Data 3.
Description: Candidate PDX models and genomic events, matched to NCI-MATCH study arms and treatments.

File Name: Supplementary Data 4
Description: The reported and potentially novel druggable alterations.

File Name: Supplementary Data 5
Description: Gene expression related genetic alterations and target arms per cancer type.

File Name: Supplementary Data 6
Description: Online resources to the represented PDX centers and models.
